# Post-tracheostomy tracheoinnominate fistula: endovascular treatment

**DOI:** 10.1590/1984-0462/2022/40/2020229

**Published:** 2021-07-07

**Authors:** Lisieux Eyer de Jesus, Eduardo Wagner Guimarães Marques da Silva, Marcos Balieiro, Karen Feldman, Samuel Dekermacher

**Affiliations:** aHospital Federal dos Servidores do Estado, Ministry of Health, Rio de Janeiro, RJ, Brazil.

**Keywords:** Tracheostomy, Brachiocephalic trunk, Hemorrhage, Endovascular procedures, Traqueostomia, Tronco braquiocefálico, Hemorragia, Técnicas endovasculares

## Abstract

**Objective:**

Tracheoinnominate fistula (TIF) is a rare and frequently lethal complication of tracheostomies. Immediate bleeding control and surgical treatment are essential to avoid death. This report describes the successful endovascular treatment of TIF in a preschooler and reviews the literature concerning epidemiology, diagnosis, prophylaxis, and treatment of TIF in pediatric patients.

**Case description:**

A tracheostomized neurologically impaired bed-ridden three-year-old girl was admitted to treat an episode of tracheitis. Tracheostomy had been performed two years before. The child used a plastic cuffed tube continually inflated at low pressure. The patient presented two self-limited bleeding episodes through the tracheostomy in a 48h interval. A new episode was suggestive of arterial bleeding, immediately leading to a provisional diagnosis of TIF, which was confirmed by angiotomography, affecting the bifurcation of the innominate artery and the right tracheal wall. The patient was immediately treated by the endovascular placement of polytetrafluoroethylene (PTFE)/nitinol stents in Y configuration. No recurrent TIF, neurological problems, or right arm ischemia have been detected in the follow-up.

**Comments:**

TIF must be suspected after any significant bleeding from the tracheostoma. Endovascular techniques may provide rapid bleeding control with low morbidity, but they are limited to a few case reports in pediatric patients, all of them addressing adolescents. Long-term follow-up is needed to detect whether stent-related vascular complications will occur with growth.

## RESUMO

**Objetivo:**

As fístulas traqueoinominadas (TIF) são complicações raras e frequentemente letais das traqueostomias (TQT). Controle imediato do sangramento e tratamento cirúrgico são essenciais para evitar a morte. Este trabalho relata o tratamento endovascular bem-sucedido de uma TIF em um pré-escolar e revisa a literatura a respeito da epidemiologia, profilaxia, diagnóstico e tratamento de TIF em pacientes pediátricos.

**Descrição do caso:**

Uma criança de 3 anos de idade, com encefalopatia, restrita ao leito e traqueostomizada havia dois anos foi internada para tratar um episódio de traqueíte. A criança usava uma cânula plástica balonada continuamente inflada com baixa pressão. A paciente apresentou dois episódios autolimitados de sangramento pela traqueostomia em um intervalo de 48 horas. Um novo episódio foi sugestivo de sangramento arterial e assumiu-se o diagnóstico provisório de TIF, confirmado através de angiotomografia, atingindo a bifurcação da artéria inominada e a parede direita da traqueia. A paciente foi imediatamente tratada pela inserção endovascular de um enxerto de politetrafluoroetileno (PTFE)/nitinol em “Y”. No seguimento, não foram encontradas recorrência de TIF, sequelas neurológicas ou isquemia do braço direito.

**Comentários:**

Deve-se suspeitar de TIF sempre que houver um sangramento significativo pelo traqueoestoma. Técnicas endovasculares possibilitam o rápido controle do sangramento com baixa morbidade, mas estão limitadas a poucos relatos de caso e pacientes pediátricos, sendo todos em adolescentes. O seguimento a longo prazo é necessário para avaliar se ocorrem complicações vasculares dos *stents* com o crescimento.

## INTRODUCTION

Tracheostomy is often indicated for children with severe chronic encephalopathy. Lethal complications are related to accidental decannulation, cannula obstruction, and tracheoinnominate fistula (TIF).

TIF is uncommon (0.6-0.7% or 1:150 adult tracheostomy),^1^ ^,^ ^2^ but presents very high mortality. Due to its relative rarity, the experience of any given surgical team is restricted, limiting early recognition and successful treatment.

Immediate bleeding control and low surgical morbidity are essential to avoid death in this group of fragile patients.

Neurologically impaired pediatric patients are a special group. Tracheostomy is frequently used as a definitive treatment. These patients are prone to late tracheostomy complications: in this group, most TIF episodes are late. Endovascular treatment of TIF may be advantageous to avoid the risk of perioperative exsanguination and the morbidity of thoracotomies. However, its use has not been reported in young children, in whom future growth must be considered. We herein describe the case of successful endovascular treatment of TIF in a preschooler and review the literature concerning epidemiology, diagnosis, prophylaxis, and treatment of TIF in neurologically impaired pediatric patients.

## CASE REPORT

A three-year-old bed-ridden girl presenting a chronic neurologic condition (Zika syndrome) was admitted to treat an episode of tracheitis. She had been submitted to tracheostomy and gastrostomy two years before to treat chronic pulmonary aspiration, recurrent episodes of pneumonia, and swallowing incoordination. A plastic cuffed tube continually inflated at low pressure was prescribed, as the child needed intermittent positive pressure ventilation.

The patient presented two self-limited bleeding episodes through the tracheostomy in a 48h interval, initially attributed to aspiration trauma. A new episode 48 hours after the initial report with bright projectile blood suggestive of arterial bleeding was then described by the mother, requiring a surgical evaluation. The patient did not show physiological instability or severe anemia.

A provisional diagnosis of TIF was made by the pediatric surgery team. An immediate angiotomography confirmed the diagnosis and revealed a TIF affecting the bifurcation of the innominate artery (IA) and the right tracheal wall, with a small pooling of contrast next to the fistula.

**Figure 1:**
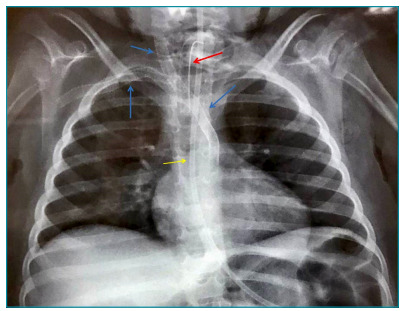
Thoracic radiography demonstrating the position of intra-arterial stents. Blue arrows: implanted polytetrafluoroethylene stents; red arrow: tracheostomy tube; yellow arrow: nasogastric tube.

The patient was treated by the endovascular placement of four expanded polytetrafluoroethylene (PTFE)/nitinol stents (Viabhan^®^) in the right carotid (5×50 and 6×100 mm) and right subclavian arteries (5×25 and 5×100 mm), crossing the transition to the IA in Y configuration (Figure 1). A stent (5×25 mm) accidentally migrated, staying next to the iliac bifurcation. This dislocated stent was not retrieved, since the team considered that removal attempts implied an unfavorable risk-benefit ratio. No neurological worsening, right arm ischemia, or complications related to the migrating stent were detected after surgery. Angiographic control, including a new angiotomography four months after surgery, showed normal blood flow in the right carotid and right subclavian arteries.

Anticoagulants were prescribed for two months (subcutaneous enoxaparin 20 mg bid). Five months after surgery, the patient remains asymptomatic, without new bleeding episodes, as well as infectious, thrombotic, or ischemic complications related to the stents.

## DISCUSSION

The most significant tracheostomy bleeding - >48 hours after the procedure - was due to TIF.^1^ As a rule, patients bleeding >10 mL from the tracheostomy stoma or cannula >48 hours after the procedure must be assumed to have a TIF until proven otherwise. The literature suggests that most cases present in the first month after tracheostomy, but this finding is questionable when it comes to neurologically impaired children submitted to definitive tracheostomy or laryngotracheal separation (LTS). In this group, most TIF episodes are late and seem to be more common (4.0-6.2% of the patients).^3^

TIF is caused by progressive erosion of the tracheal wall, finally communicating with the adjacent mediastinal arterial vessels, most commonly IA, which is located between the trachea and the sternum in the superior mediastinum. Pressure from a constantly inflated balloon, especially at high pressures, or constant contact with the tip of the cannula cause the problem.^1^ Necrosis, chronic inflammation, granulation tissue, and scarring are the hallmarks of the condition.^4^ ^,^ ^5^ Thoracic deformities (including scoliosis), a shortened distance between the sternum and the vertebral column,^3^ and abnormal neck positioning/opisthotonus add to the risk.^6^ Vascular abnormalities, tracheitis, a shortened neck, and placement of the tracheostomy under the 4^th^ tracheal ring are also related to TIF.

One-third to one-half of the cases present preliminary episodes of self-limited bleeding through the tracheostomy (“sentinel bleeding”), followed by severe bleeding causing hypovolemic shock, exsanguination, and/or asphyxia. A few patients have shown pulsating cannula.^1^ Bronchoscopy/tracheoscopy are controversial in this situation, as they imply manipulation of the trachea and potential dislocation of the cannula, with the risk of losing hemostasis during the process,^7^ being only indicated if it turns to be the only way to compress the fistula while awaiting surgery.^8^ ^,^ ^9^ The diagnosis may be confirmed by angiotomography in stable patients, but the exam might only demonstrate the crossing of the IA located over the inflated balloon or cannula tip and/or local irregularity suggesting inflammation. Intraoperative arteriography remains the gold standard to date.

In most cases, the vascular defect is small,^1^ but it may reach 10 mm.^7^ ^,^ ^8^ ^,^ ^9^ ^,^ ^10^ ^,^ ^11^ Bleeding can be controlled by hyperinsufflation of the cannula balloon in 85% of cases.^1^ The cannula should not be removed and must be stabilized in position after bleeding control. Direct digital compression of the IA against the sternum through a cutaneous incision just over the manubrium or through the tracheostome, in parallel to the trachea or the ventilation tube, may work for adults,^1^ but are difficult or impossible in children due to the small caliber of the pediatric trachea and thoracic inlet.^9^

Classical treatment requires emergency sternotomy/thoracotomy. Perioperative shock and exsanguination are common, and the survival rate has been reported as 25% after open surgery (versus zero after expectant treatment).^12^ Proximal and distal control of the involved vessels is essential before dissecting the fistula. Suprasternal transverse incisions^3^ or limited upper sternotomies with or without “collar” incision^13^ offer lower morbidity and quick access, but exposure is limited, especially in children. Thoracotomy on extracorporeal membrane oxygenation (ECMO) and tracheal occlusion have been used to treat an adolescent in Japan,^14^ but this option is rarely available.

Direct closure of the fistula is inadequate due to the inflammation of arterial and tracheal walls. The involved structures are unable to hold sutures during surgery, or pseudoaneurysms follow, presenting as brisk bleeding due to early recurrence. Most authors recommend ligation and excision of the involved IA segment, reporting no neurological sequelae, provided the presence of a patent circle of Willis.^1^ ^,^ ^8^ ^,^ ^10^ Pericardial and vascular patches, as well as local and extra-anatomical bypasses with PTFE grafts, are options, limited by complexity and morbidity, including pseudoaneurysm secondary to graft infection. The tracheal defect should be sutured and covered with a patch of healthy vascularized tissue. Whenever possible, the balloon should be inflated distal to the tracheal suture.

Endovascular methods show many advantages when compared to emergent thoracotomy, allowing direct, precise, and immediate bleeding control with lower morbidity and no risk of inducing uncontrolled bleeding during exposure. Endovascular treatment of TIF in children was first reported in 2005, using coil embolization of the IA, without neurological or right arm ischemic sequelae, comparable to IA segmental resection, without the morbidity of sternotomy and vascular dissection.^12^ Successful use of self-expanding stents has also been reported in adolescents, with limited follow-ups (14 mo).^15^

To the best of our knowledge, our case is the first to report the endovascular treatment of a complex TIF affecting the IA bifurcation in a young child. Another case has been reported of endovascular stent implantation in a four-year-old child to treat a pseudoaneurysm secondary to previous treatment of a complex congenital tracheal stenosis with a pericardial patch. This patient died four months later from unrelated causes, with no signs of vascular complications.^16^

Stent grafting in children is arguable, given the potential stent-body size mismatch as the child grows. Considering the lack of long-term follow-up data in the literature, we do not know if future problems may arise, requiring the replacement of stents or the planning of a bypass due to the child’s growth. This procedure may not be necessary even if the stents become disproportional due to the development of collateral vessels. We should, however, bear in mind the survival prognosis and life limitations of severely neurologically impaired patients, as defined by their primary condition. Also, the rapid control of a bleeding emergency with minimal morbidity and mortality is advantageous. Even with growth, considering primary and compensating collaterals, especially with an adequate circle of Willis, the diameter of the stent probably will not be related to ischemic problems. If needed, elective vascular bypasses can be planned in the future. Infectious complications have not been reported so far, despite the placement of a prosthetic stent in a theoretically contaminated surgical field, but antibiotic coverage is needed. We recognize that information about this technique is scarce in the literature even for adults, with limited follow-ups.

Some authors suggest that, in high-risk patients (neurologically impaired patients presenting severe deformities with potential IA compression), prophylactic arterial ligation prior to tracheostomy should be adopted after radiological proof of efficient collateral flow through the right carotid artery territory.^3^ Granulation tissue associated with pulsation and red pulsating ulcers in routine bronchoscopic follow-up or the extreme proximity between cannula tip and IA are warning signs in patients with chronic tracheostomy.^17^
